# 异基因造血干细胞移植治疗慢性粒-单核细胞白血病的临床结局

**DOI:** 10.3760/cma.j.cn121090-20231007-00160

**Published:** 2024-01

**Authors:** 晓辉 张, 建英 周

**Affiliations:** 北京大学人民医院、北京大学血液病研究所、国家血液系统疾病临床医学研究中心、造血干细胞移植北京市重点实验室，北京 100044 Peking University People's Hospital, Peking University Institute of Hematology, National Clinical Research Center for Hematologic Disease, Beijing Key Laboratory of Hematopoietic Stem Cell Transplantation, Beijing100044, China

**Keywords:** 慢性粒-单核细胞白血病, 异基因造血干细胞移植, 临床结局, Chronic myelomonocytic leukemia, Allogeneic hematopoietic stem cell transplantation, Clinical outcomes

## Abstract

慢性粒-单核细胞白血病（CMML）是一种来源于骨髓造血干细胞的克隆性疾病，预后较差。异基因造血干细胞移植（allo-HSCT）是可治愈CMML的方法之一。患者移植后结局受疾病特征和患者共病等多种因素影响。根据现有预后分层系统，筛选出适合移植的CMML患者并早期移植，有利于其长期生存。医生可根据新开发的移植预后模型评估移植后CMML患者的生存情况并作出针对性的医疗决策。

## Abstract (en)

Chronic myelomonocytic leukemia (CMML) is a clonal disease derived from bone marrow hematopoietic stem cells, with a poor prognosis. Allogeneic hematopoietic stem cell transplantation (allo- HSCT) is one of the curable methods for CMML. The outcome of patient transplantation is influenced by various factors such as disease characteristics and comorbidities. Based on the existing prognostic stratification system, screening suitable CMML patients for transplantation and early transplantation is beneficial for their long-term survival. Doctors can evaluate the survival status of CMML patients after transplantation based on the newly developed transplant prognosis model and make targeted medical decisions.

慢性粒-单核细胞白血病（CMML）为来源于骨髓造血干细胞的克隆性疾病，其特征为外周血单核细胞计数增多，骨髓细胞形态学检查可见骨髓增生异常综合征（MDS）和骨髓增生性肿瘤（MPN）特征重叠[1]。CMML患者向急性髓系白血病（AML）转化的风险较高，预后较差，根据不同分层，生存期数月至数年不等[2]–[3]。allo-HSCT是目前公认的治愈方法之一[1],[4]–[5]，但移植后患者结局受多种因素影响。

一、基于预后分层系统的allo-HSCT治疗

欧洲白血病网（ELN）和欧洲血液学会（EHA）2018年发表的CMML诊断及治疗指南中推荐了以下5种预后分层系统：CMML临床/分子预后积分系统（CPSS- mol）、梅奥分子模型（MMM）、骨髓增生异常法国分组（GFM）、MD安德森预后积分（MDAPS）和CMML特异性预后积分系统（CPSS）[1]。其中最常用于评估allo-HSCT治疗患者选择的预后分层系统为CPSS[6]–[7]。适合移植的CMML患者包括：CPSS评分为中危-2或高危的患者，或CPSS评分为中危-1或低危但合并高危细胞遗传学异常、持续原始细胞升高、严重的血细胞减少、输血依赖或伴有ASXL1、NRAS、RUNX1和SETBP1等基因突变者[1],[4]。没有人类白细胞抗原（HLA）全相合供者时可考虑包括单倍体供者在内的替代供者[8]–[9]。不适合移植的患者可根据病情及WHO分型接受去甲基化药物（HMA）、羟基脲、对症支持等治疗[1],[4]。

二、allo-HSCT的疗效优势

Pophali等[10]依据患者年龄及预后分层，将移植与非移植CMML患者按照1∶1进行倾向得分匹配（PSM）分析，结果显示移植组患者的中位总生存（OS）期显著长于非移植组（40个月对23个月，*P*＝0.004）；将未转化为AML的CMML患者（CP-CMML）按照1∶1进行PSM分析，与非移植组相比，移植组仍具有显著的生存优势（中位OS期40个月对21个月，*P*＝0.002），转化为AML的CMML患者（BT-CMML）也可从移植中获益（中位OS期22个月对3个月，*P*＝0.0001）。因此研究者认为allo-HSCT可使CMML患者生存明显获益，尤其在CP-CMML患者中，该研究亦强调早期接受allo-HSCT的重要性。

三、影响allo-HSCT疗效的因素

不同研究均表明，诊断后早期（1年内）行allo-HSCT的患者无复发生存（RFS）率和OS率都高于晚期（1年后）移植者[11]–[13]。

不良染色体核型及基因突变也会影响allo-HSCT结局。按照西班牙细胞遗传学分层将接受allo-HSCT治疗的CMML患者分为低危、中危和高危组[14]，移植后3年OS率分别为56.7%、12.5%、0%（*P*＝0.01）。与低危组相比，中危和高危组与不良预后显著相关（*HR*＝3.5，*P*＝0.01；*HR*＝3.9，*P*＝0.05）。Eissa等[15]的研究亦表明，CMML患者的异常核型与移植后死亡率相关，伴高危细胞遗传学异常的患者移植后总体死亡率及非复发死亡（NRM）率升高（*HR*＝2.07，*P*＝0.02；*HR*＝2.83，*P*＝0.008），RFS降低。其他研究也表明，具有高危细胞遗传学异常（*HR*＝3.77，*P*＝0.0002；*HR*＝1.88，*P*＝0.01）以及移植前微小残留病（MRD）阳性的细胞遗传学异常（*HR*＝2.55，*P*＝0.007；*HR*＝1.65，*P*＝0.02）的患者移植后复发率及总体死亡率明显升高，相应的移植后患者的生存期缩短[16]。

一项纳入185例接受allo-HSCT的CMML患者的研究，筛选出4种可影响移植后患者生存期的高危基因突变（HRM）：ASXL1、KRAS、SF3B1、ZRSR2，根据基因突变数量将患者分为No HRM、1-2 HRM、3-4 HRM三组，移植后6年OS率分别为59%、34%、14%（*P*<0.001）[17]。CIBMTR研究分析了CMML患者基因突变谱对allo-HSCT结局的影响，结果显示突变基因数量与移植相关死亡（TRM）相关（*HR*＝1.17，*P*＝0.0031）[18]。Woo等[16]研究发现，移植前总突变负荷较高（≥10个突变）以及>4个表观遗传调控相关基因突变的患者移植后复发风险增高（*HR*＝1.5，*P*＝0.02；*HR*＝1.5，*P*＝0.003）。

MD安德森癌症中心的一项回顾性研究分析了移植前治疗方案对患者移植后生存的影响[19]，与其他治疗方法相比，接受HMA治疗的患者移植后3年复发率较低（22%对35%，*P*＝0.03）、PFS率较高（43%对27%，*P*＝0.04），对OS率则无显著影响（45%对39%，*P*＝0.22）。

移植前疾病状态对患者的移植结局也有影响。移植前达到完全缓解（CR）的患者具有较低的NRM，RFS与OS期较长；移植前没有达到CR的患者具有较低的OS率，中性粒细胞及血小板植入不良的发生率也较高[11],[20]。

不同研究团队均发现移植前造血干细胞移植相关合并症指数（HCT-CI）较高的患者移植后NRM及总体死亡率均明显升高、生存期缩短[12],[15],[21]。

欧洲血液和骨髓移植小组比较了清髓性预处理方案（MAC）低剂量预处理方案（RIC）对allo-HSCT后CMML患者生存期的影响，结果表明两组之间移植后1年NRM（*P*＝0.53）、4年RFS率（*P*＝0.37）、OS率（*P*＝0.89）和复发率（*P*＝0.91）均无显著差异[11]。根据预处理方案中是否有全身放射治疗（TBI）将患者分为non-TBI与TBI组，移植后1年NRM率（*P*＝0.66），4年RFS率（*P*＝0.51），OS率（*P*＝0.92）和复发率（*P*＝0.82）也不存在显著差异。

此外，有研究表明移植时伴脾脏肿大患者的OS（*HR*＝0.48，*P*＝0.04）和无事件生存期（EFS）（*HR*＝0.44，*P*＝0.02）明显低于移植时无脾脏肿大的患者，移植时伴脾脏肿大的患者移植后CIR升高（47%对25%，*P*＝0.07），2年OS率降低（28%对52%，*P*＝0.03）[22]。然而，也有研究表明allo-HSCT前脾脏肿大对移植后CMML患者生存及复发无影响[23]。因此，移植前脾脏肿大对移植后CMML患者预后的影响尚需要进一步大样本研究证实。

其他可能影响移植后CMML患者生存期的因素包括：移植前骨髓原始细胞<5%（*HR*＝0.36，*P*＝0.04），移植后慢性移植物抗宿主病（GVHD）（*HR*＝0.15，*P*＝0.001）与移植后较长的PFS相关[19],[24]。移植后发生Ⅱ～Ⅳ级急性GVHD（*HR*＝2.7，*P*＝0.01）的患者PFS期较短。

四、allo-HSCT后并发症

CMML患者allo-HSCT常见并发症包括急性GVHD、慢性GVHD、感染、特发性肺炎、器官衰竭、出血、血栓性微血管病以及肝窦阻塞综合征等[20],[25]–[26]。目前暂无文献报道CMML患者allo-HSCT后存在特异并发症。

五、allo-HSCT治疗CMML患者的预后模型

1. 现有预后分层系统对移植后结局的预测价值：研究者基于现有的5种CMML预后分层系统对移植后患者的生存期进行分析，结果发现，与只包含临床变量的CPSS及MDAPS分层系统相比，包含基因突变信息的CPSS-mol、MMM以及GFM分层系统对移植后患者的生存期预测更具有指导意义。

CIBMTR回顾性研究发现CPSS和CPSS- mol分层系统的不同分层与患者的OS、无病生存期（DFS）、TRM以及复发相关[18]。CPSS评分中危-2及高危是OS的不良预后因素（*HR*＝1.46，*P*＝0.0494；*HR*＝3.22，*P*＝0.0004），此外高危评分与DFS（*HR*＝2.24，*P*＝0.012）及复发（*HR*＝2.73，*P*＝0.012）显著相关。CPSS-mol评分高危与较差的OS（*HR*＝2，*P*＝0.0079）和DFS（*HR*＝1.73，*P*＝0.024）相关，与CPSS-mol高危评分相比，中危-1和中危-2评分与较低的TRM相关（*HR*＝0.32，*P*＝0.0078）。

MDAPS及CPSS预后分层系统中高危患者移植后复发率较高（*HR*＝9.41，*P*＝0.005；*HR*＝14.3，*P*＝0.01）[16]。其他研究团队的结论与之类似[15],[21]。

2. allo-HSCT治疗CMML结局预测模型：德国一项多中心回顾性研究[27]通过多因素分析发现4种与移植后患者生存相关的独立危险因素：ASXL1/NRAS基因突变（*HR*＝1.63）、骨髓原始细胞>2%（*HR*＝1.70）和共病指数增加（*HR*＝1.16）。据此总结出CMML移植评分：ASXL1/NRAS基因突变（各4分），骨髓原始细胞>2%（4分），共病指数每增加一个1分。CMML移植评分（范围0～20分）可以预测CMML患者移植后的OS和NRM率。该评分系统划分了5个风险组，0～1分组的5年OS率、NRM分别为81%、5%，2～4分组分别为49%、22%，5～7分组分别为43%、31%，8～10分组分别为31%、46%，>10分组分别为19%、51%（*P*<0.001，*P*<0.001）。该评分纳入患者的基因突变和临床信息，具有预后评估价值并有助于个性化咨询。

一项国内27家中心参与、纳入218例CMML患者的多中心研究[28]结果显示，年龄>60岁（1分）、移植之前骨髓原始细胞比例>10%（1分）、初诊WBC>32×10^9^/L（1分）、初诊HGB≤120 g/L（1分）、慢性GVHD（2分）是影响患者移植后生存期的独立危险因素，据此构建ABLAG预后模型并将患者分为低危组（0～1分）、中危组（2～3分）和高危组（4～6分），三组移植后3年OS率分别为93.3%、78.9%、51.6%（*P*<0.001），NRM分别为6.1%、17.3%、36.8%（*P*<0.001）。与CPSS、MDAPS、CPSS-mol、MMM及GFM模型比较，ABLAG预后模型具有更好的净获益。

综上，allo-HSCT是目前唯一可治愈CMML的方法，适合移植的患者确诊后应尽早移植，有利于获得长期生存。在移植之前，根据病情可接受HMA治疗。移植前获得CR可减少移植后复发、延长生存期。移植预处理方案可根据患者的身体状况选择MAC或RIC，不同预处理方案不影响移植后生存期。医生可根据新开发的ABLAG型预后模型评估CMML患者移植后的生存情况并作出针对性医疗决策。
